# Nearshore wave buoy data from southeastern Australia for coastal research and management

**DOI:** 10.1038/s41597-023-02865-x

**Published:** 2024-02-12

**Authors:** Michael A. Kinsela, Bradley D. Morris, Timothy C. Ingleton, Thomas B. Doyle, Michael D. Sutherland, Neil E. Doszpot, Jeff J. Miller, Stephen F. Holtznagel, Mitchell D. Harley, David J. Hanslow

**Affiliations:** 1 Water, Wetlands and Coasts Science, Environment and Heritage, Department of Climate Change, Energy, the Environment and Water, NSW Government, Lidcombe, Australia; 2 School of Environmental and Life Sciences, University of Newcastle, Callaghan, Australia; 3 Water Research Laboratory, School of Civil and Environmental Engineering, UNSW, Manly Vale, Australia

**Keywords:** Natural hazards, Physical oceanography

## Abstract

Wind wave observations in shallow coastal waters are essential for calibrating, validating, and improving numerical wave models to predict sediment transport, shoreline change, and coastal hazards such as beach erosion and oceanic inundation. Although ocean buoys and satellites provide near-global coverage of deep-water wave conditions, shallow-water wave observations remain sparse and often inaccessible. Nearshore wave conditions may vary considerably alongshore due to coastline orientation and shape, bathymetry and islands. We present a growing dataset of *in-situ* wave buoy observations from shallow waters (<35 m) in southeast Australia that comprises over 7,000 days of measurements at 20 locations. The moored buoys measured wave conditions continuously for several months to multiple years, capturing ambient and storm conditions in diverse settings, including coastal hazard risk sites. The dataset includes tabulated time series of spectral and time-domain parameters describing wave height, period and direction at half-hourly temporal resolution. Buoy displacement and wave spectra data are also available for advanced applications. Summary plots and tables describing wave conditions measured at each location are provided.

## Background & Summary

Reliable measurements of nearshore wave conditions are essential for calibrating, validating and improving shallow-water wave transformation models^1,2^, which are used by coastal scientists and engineers to define local wave climates and extremes, drive shoreline response and coastal evolution models, and investigate and predict coastal processes, dynamics and hazards^3–5^. The local wave conditions impacting a beach or rocky shore may vary considerably from adjacent coasts for the same offshore wave conditions^1^, due to alongshore variation in coastline orientation, the presence of coastal islands, shoreline shape, and seabed bathymetry and texture across the wave shoaling zone (shoreface). Ocean wave buoy networks^6^, satellite altimeters^7^, and deep-water wave models^8^ (some with data assimilation) provide historical and forecast ocean wave data at a global scale, however, shallow-water wave observations are rarely available. This is a critical knowledge gap for wind-wave^9^ and coastal geoscience and engineering^10^ research in Australia.

Cost, resource, logistical and time limitations mean that it is often infeasible to deploy a wave measurement device (e.g., wave buoy) to study local wave conditions at sites of interest for coastal research or management. A shallow-water wave model is usually applied to simulate nearshore wave conditions based on measured or modelled deep-water wave data^1,2^. The limited availability of nearshore wave observations means that shallow-water wave models may be subject to limited or no calibration and validation in practice, despite having crucial importance in simulating coastal processes and hazards of interest^3–5^. This is most problematic in settings with complex seabed geomorphology, where modelled wave conditions may be more sensitive to the design (including bathymetry and bed friction) of shallow-water wave models. Without nearshore wave observations covering different shore (sandy, gravel, rocky) and seabed (sediment, reef, mixed) types, shoreface slopes, and ocean wave exposure, the accuracy of shallow-water wave modelling in practice often remains poorly quantified.

**Fig. 1:**
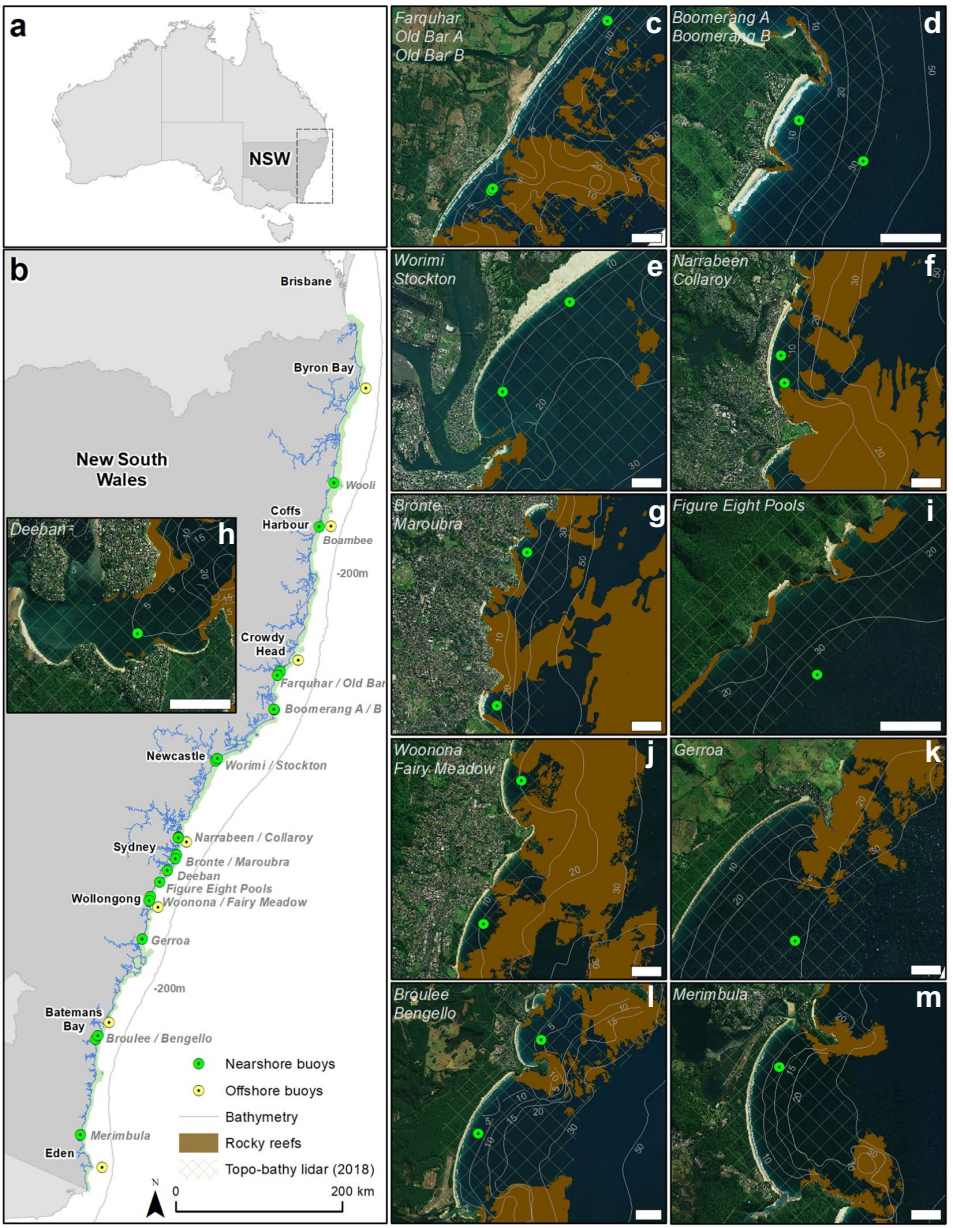
The 20 nearshore wave buoy deployments along the coast of New South Wales, Australia (**a,b**) that were completed or in progress at 31/8/2023 (Tables 1–3). Locations of seven long-term offshore wave buoy deployments^13^ (65–80 m water depths) are also shown. Site location maps (**c**–**m**) showing aerial imagery, high-resolution lidar topography-bathymetry^14^ and seabed landforms^15^ mapping, with general bathymetry, are provided for 18 locations for which final data is available and included in the dataset (Tables 1–2). All site maps are oriented north-up with 1-km scale bars (white) and the names of deployments shown in each panel are listed from north to south at top left.

We present a growing dataset of *in-situ* shallow-water (<35 m depth) wave observations along the coast of New South Wales (NSW), southeastern Australia (Fig. 1). The ongoing program of short-term (months to years) wave buoy deployments covers a range of coastal settings and geomorphology, including those where infrastructure and properties are known to be at risk from coastal erosion and wave inundation^11,12^. Since March 2016, 81 moored wave buoy deployments have been carried out at 20 locations (Fig. 1), collecting over 7,000 days of half-hourly wave data as of 30 November 2023 (Tables 1–3). The dataset captures both fair-weather and storm-wave conditions (significant wave height, *H*_*m0*_ > 3 m), including a storm in June 2016^12^ during which one wave buoy measured *H*_*m0*_ approaching 7 m, and multiple *H*_*m0*_ observations exceeding 5 m at other locations to date (Tables 1, 2). We provide commonly used time series data, including spectral and time-domain parameters for wave height, period and direction at half-hourly temporal resolution, with plots and tables summarising measured wave conditions at each location. Parameter data from active wave buoy deployments can also be viewed and downloaded in real time. For advanced applications, wave buoy displacement data and wave spectra data are also made available through the Australian National Wave Archive (see Data Records).

**Table 1:** Completed nearshore wave buoy deployments using Datawell buoys listed from north to south (Fig. 1).

Location	Geoposition (centroid)	Start date	End date	Data days (% success)	Peak H_m0_ (m)	Instruments (deployments)
Farquhar	–31.93132 152.63301 –12.3 m	16/8/2018	4/12/2018	109 (99.5)	4.20	Datawell-G4 (3)
Old Bar A	–31.98063 152.59317 –12.5 m	16/8/2018	4/12/2018	109 (99.1)	3.55	Datawell-G4 (3)
Boomerang B	–32.34791 152.55802 –33.0 m	21/3/2019	18/6/2019	80 (89.9)	3.87	Datawell-G4 (2)
Narrabeen	–33.71880 151.30467 –12.4 m	2/3/2016	17/5/2016	76 (100)	2.61	Datawell-G4 (3)
Collaroy	–33.72705 151.30596 –13.2 m	2/3/2016	17/5/2016	76 (100)	1.98	Datawell-G4 (3)
Collaroy 2	–33.72691 151.30605 –13.3 m	3/6/2016	21/7/2016	29 (60.4)	6.91	Datawell-G4 (2)
Bronte	–33.90477 151.27455 –13.6 m	11/8/2016	26/10/2016	55 (72.4)	3.05	Datawell-G4 (2)
Maroubra	–33.94992 151.26297 –13.6 m	11/8/2016	26/10/2016	72 (94.7)	4.10	Datawell-G4 (2)
Figure 8 Pools	–34.19984 151.05012 –30.0 m	6/12/2016	18/12/2017	370 (98.1)	5.66	Datawell-G4 (10)
Woonona	–34.35491 150.92628 –13.6 m	5/6/2017	30/10/2017	147 (100)	3.27	Datawell-G4 (4)
Fairy Meadow	–34.39710 150.91225 –14.5 m	5/6/2017	30/10/2017	114 (77.6)	3.65	Datawell-G4 (4)
Gerroa	–34.81436 150.80965 –30.5 m	13/6/2018	27/7/2018	39 (88.6)	3.17	Datawell-G4 (1)
All locations		2/3/2016	18/6/2019	**1276**	6.91	Datawell-G4 (39)

Geopositions are the latitude and longitude (WGS84) of the deployment centroid and corresponding water depth (m AHD)^14^. Data days are the number of days of successful data collection during the deployment period (also shown as % deployment duration). Peak H_m0_ is the highest significant wave height measured (excluding *Qflag* 4). The instruments used and number of buoy deployments are provided for each location.

**Table 2:** Completed nearshore wave buoy deployments using Spotter buoys listed from north to south (Fig. 1).

Location	Geoposition (centroid)	Start date	End date	Data days (% success)	Peak H_m0_ (m)	Instruments (deployments)
Farquhar	–31.93132 152.63301 –12.3 m	4/12/2018	12/3/2019	98 (100)	3.57	Spotter-v1 (1)
Old Bar B	–31.98135 152.59238 –13.0	3/10/2018	11/3/2019	158 (99.4)	3.15	Spotter-v1 (3)
Boomerang A	–32.34228 152.54684 –11.4 m	21/3/2019	4/6/2019	75 (100)	2.80	Spotter-v1 (1)
Boomerang A2	–32.34284 152.54759 –13.3 m	24/7/2019	20/5/2020	301 (100)	5.20	Spotter-v1 (2)
Boomerang B2	–32.34831 152.55781 –32.8 m	24/7/2019	16/1/2020	173 (98.3)	4.35	Spotter-v1 (2)
Worimi	–32.87583 151.82245 –13.6 m	6/12/2019	26/2/2020	81 (98.8)	4.35	Spotter-v1 (1)
Stockton	–32.90213 151.79869 –13.2 m	6/12/2019	9/4/2021	490 (100)	6.06	Spotter-v1 (4)
Collaroy 3	–33.72644 151.30695 –13.6 m	21/10/2019	31/3/2020	109 (67.3)	4.19	Spotter-v1 (1)
Collaroy 4	–33.72703 151.30695 –15.2 m	31/3/2020	2/11/2021	581 (100)	4.03	Spotter-v1 (2)
Collaroy 5	–33.72657 151.30665 –14.5 m	21/12/2021	31/8/2023	617 (100)	4.88	Spotter-v1 (3)
Deeban	–34.07870 151.15361 –6.1 m	30/6/2021	7/12/2021	160 (100)	1.27	Spotter-v2 (1)
Deeban 2	–34.0784 151.15377 –6.1 m	1/3/2022	16/11/2022	260 (100)	2.14	Spotter-v2 (1)
Broulee	–35.84837 150.18879 –13.2 m	10/11/2020	24/8/2021	287 (100)	3.65	Spotter-v1 (2)
Broulee 2	–35.84841 150.18878 –13.2	19/11/2021	24/11/2022	370 (100)	5.35	Spotter-v1 (2)
Bengello	–35.87995 150.16120 –12.8 m	10/11/2020	9/6/2023	941 (100)	6.33	Spotter-v1 (3) Spotter-v2 (2)
Merimbula	−36.90860 149.91653 −13.1 m	16/11/2020	10/5/2021	137 (78.3)	3.64	Spotter-v1 (1)
Merimbula 2	−36.90841 149.91662 −13.0 m	10/5/2021	23/11/2022	562 (100)	6.28	Spotter-v1 (3)
All locations		16/8/2018	31/8/2023	**5400**	6.33	Spotter-v1 (31) Spotter-v2 (4)

Geopositions are the latitude and longitude (WGS84) of the deployment centroid and corresponding water depth (m AHD)^14^. Data days are the number of days of successful data collection during the deployment period (also shown as % deployment duration). Peak H_m0_ is the highest significant wave height measured (excluding *Qflag* 4). The instruments used and number of buoy deployments are provided for each location.

**Table 3:** In-progress nearshore wave buoy deployments as at 30 November 2023 listed from north to south (Fig. 1).

Location	Geoposition (centroid)	Start date	End date	Data days	Peak H_m0_ (m)	Instruments (deployments)
Wooli	−29.86938 153.27617 −13.0 m	21/2/2023	—	273	3.27	Spotter-v2 (2)
Boambee	−30.33580 153.12412 −13.4 m	22/2/2023	—	281	3.72	Spotter-v1 (2)
Collaroy 6	−33.72645 151.30682 −14.5 m	31/8/2023	—	91	2.55	Spotter-v1 (1)
Deeban 3	−34.07838 151.15412 −6.0 m	3/4/2023	—	241	1.22	Spotter-v2 (1)
Bengello 2	−35.88000 150.16108 −12.8 m	9/6/2023	—	174	3.68	Spotter-v3 (1)
All locations		21/2/2023	30/11/2023*	1060*	3.72*	Spotter-v1 (3) Spotter-v2 (3) Spotter-v3 (1)

Geopositions are the latitude and longitude (WGS84) of the deployment centroid and corresponding water depth (m AHD)^14^. Data days are the number of days of data collection during the deployment period. Peak H_m0_ is the highest significant wave height measured (excluding *Qflag* 4). The instruments used and number of buoy deployments are provided for each location.

The nearshore wave data will be of interest to wind-wave and coastal researchers in Australia and abroad, providing rare observation data for developing and evaluating shallow-water wave models for local or global applications. Data from seven long-term deep-water wave buoys spanning the study area (Fig. 1) are available separately^13^ and provide regional wave climate information and ocean wave boundary conditions for modelling. High-resolution (5 m grid) seamless coastal topography-bathymetry data^14^ and seabed landforms (sediment, rocky reef) mapping^15,16^ are also available separately from state-wide airborne lidar surveys reaching 30 m water depth on average^17^ (Fig. 1), and in deeper water where multibeam echosounder surveys have been completed^16,17^ (see Usage Notes). The wave buoy data will also interest researchers and applied engineers studying coastal processes, dynamics and hazards. Nearshore wave buoy deployments adjacent to multi-decadal coastal monitoring sites at Moruya (Bengello) Beach^18^ and Collaroy-Narrabeen Beach^19^ (Fig. 1) are ongoing, and together, provide community testbeds for international coastal modelling studies^20–22^.

Our motivations are to improve the understanding and prediction of coastal wave conditions in southeast Australia, to provide nearshore wave data for coastal processes research and hazard management studies^23,24^, and to advance shallow-water wave and coastal modelling methods globally through the provision of essential observation data. The nearshore wave buoy data are also being used to improve a regional-scale high-resolution coastal wave model covering the NSW coast^25,26^. Local-scale wave data are increasingly needed in NSW and globally as community exposure to coastal hazards increases with both development and climate change pressures^11,20^. Nearshore wave buoy data collected in our program have already been applied in coastal processes research^27^ and coastal risk management studies^28^, and to develop early warning systems for rocky shore wave hazards^29^ and storm impacts on sandy beaches^30^.

## Methods

### Deployment locations and durations

The NSW coast is microtidal, wave-dominated and is situated on a passive tectonic continental margin that is unusually steep and narrow by global comparison^31^, resulting in low attenuation of wave energy across the mid to outer continental shelf^32^. The coastline trends NNE to SSW (Fig. 1) with regional coastline orientation controlled by the geological framework of the margin and local variations by embayment geometry and sediment availability^31^. Coastal embayments are generally broader and shallower in the north and narrower and deeper in the south and contain sandy bay-barrier beaches extending between sections of coastal cliffs and rocky headlands^33^. Just over half of the 2,000 km ocean coastline is sandy beaches, with nearshore wave conditions influencing the orientation of sandy shorelines^31^.

The ocean wave climate is moderate-high energy by global standards, with long-term deep-water wave buoy records indicating mean significant wave height and period of 1.6 m and 8 s in the central NSW region^34^. The wave climate has a mild seasonal bias, being more energetic in austral winter months than austral summer, and experiences inter-annual to inter-decadal variability associated with climate cycles^34,35^. The directional wave climate varies along the coast, with the north experiencing more easterly wave directions compared to the south^36^. Storm wave conditions are influenced by a range of synoptic systems that are characterised by their origins and behaviour^37,38^, and deep-water significant wave heights up to 9 m and maximum wave heights over 18 m have been recorded by a network of seven long-term offshore wave buoy deployments^13^ (Fig. 1). A significant wave height threshold of 3 m is typically used to distinguish ‘storm’ from ‘fair-weather’ wave conditions^37^.

We have made 81 temporary moored wave buoy deployments at 20 nearshore locations in water depths <35 m along the NSW coastline (Fig. 1), from March 2016 to November 2023, and data collection is ongoing. Tables 1–3 provide details of the deployment positions, durations, data records and instruments used at each nearshore location, ordered from north to south for comparison with Fig. 1. The locations have been chosen based on four criteria: (1) sample a variety of coastal settings and bathymetric complexity; (2) capture concurrent data in nearby locations with contrasting wave exposure; (3) collect data at locations with known coastal hazard risks and/or active coastal process studies; and (4), complement other field operations such as multibeam echosounder seabed mapping^17^.

**Fig. 2:**
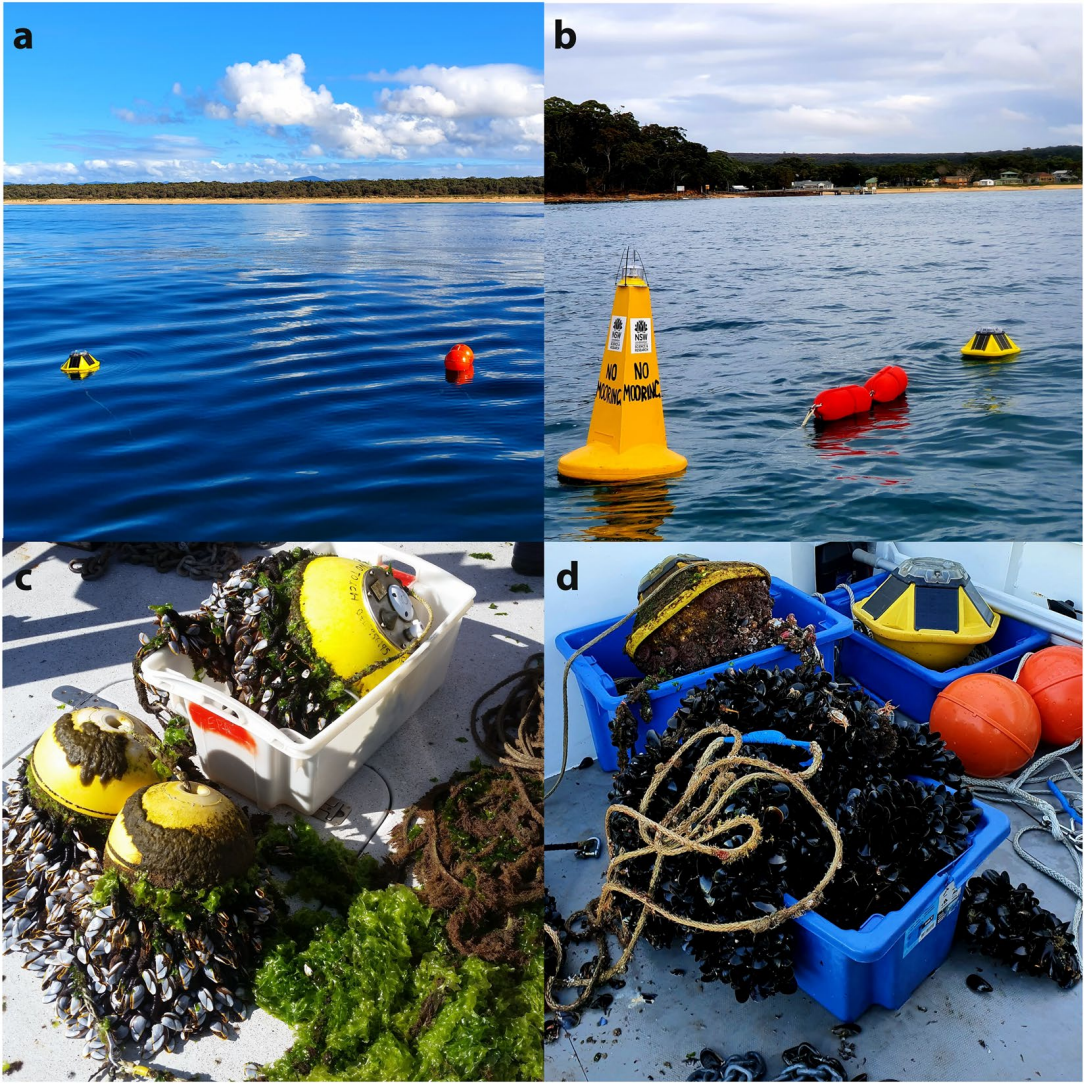
(**a**) Spotter buoy on a paired-float mooring at Bengello; (**b**) Spotter buoy on a high-visibility mooring at Deeban; (**c**) heavy fouling of a Datawell buoy and mooring at Boomerang B with goose barnacles (*Lepas pectinata*) and green algae (*Ulva sp*.); (**d**) heavy fouling of a Spotter buoy and mooring at Merimbula with blue mussels (*Mytilus galloprovincialis*). See Fig. 1 for deployment locations.

We collect nearshore wave data using small translational wave buoys deployed on temporary moorings in shallow coastal waters (Fig. 2). Deployments are placed either outside the surf zone near the base of the upper shoreface (e.g., 12 m water depth) or around the lower shoreface-inner continental shelf transition (e.g., 30 m water depth). The duration of buoy deployments varies depending on data requirements and operational considerations. For example, a brief 5-week deployment at Gerroa supported a coastal process study there^27^, while 12-month and 16-month deployments at Figure Eight Pools and Stockton Beach were intended to establish the relationship between offshore and nearshore wave conditions for hazard investigations^28,29^ (Tables 1, 2). The longest deployments are adjacent to multi-decadal beach survey datasets at Moruya (Bengello) Beach^18^ and Collaroy-Narrabeen Beach^19^ and are ongoing. Deployments are visited for servicing at regular intervals (e.g., 5 weeks for Datawell buoys and 4-6 months for Spotter buoys) to remove biofouling or replace the wave buoy and mooring with fresh equipment, and to retrieve onboard recorded data. The number of service deployments at each location is shown in Tables 1–3, and details of service deployments are provided in the readme file of each deployment data package. See Technical Validation for further details on erroneous data, data loss and omission.

### Wave buoy instruments

Two types of directional GPS/GNSS (hereafter GNSS for brevity) wave buoys have been used in the program: Datawell Waverider DWR-G4^39^ (Table 1) and Sofar Ocean Spotter^40^ (Tables 2, 3). Both instruments are small-format buoys that measure the wave field by recording their horizontal and vertical translation (displacement) using a satellite positioning receiver, rather than by measuring buoy motion and orientation physically using onboard mechanical instruments, such as accelerometers, gyros and compass. Key differences between the Datawell and Spotter buoys include the shape, fabrication materials and weight of their hulls, power systems, data storage and transmission, satellite positioning networks, and the frequency at which they record their displacement.

The Datawell buoy^39^ has a spherical steel hull of 0.40 m diameter (0.46 m with rubber fender) and weighs 17 kg as deployed with batteries and ballast chain. It operates on a battery power system with a battery life of around 30 days and thus require service visits every 4–5 weeks to maintain a continuous data record. The satellite receiver unit measures displacement using the Doppler shift of the satellite data signal. Displacement data are recorded continuously at 1.28 Hz, resulting in 2304 displacement records per half-hour sampling period, which are stored on a compact flash data card^41^. Real-time data transmission on our DWR-G4 units was limited to HF radio, which was rarely used to conserve batteries.

The Spotter buoy^40^ has a pentagonal upper/spherical lower plastic hull of 0.42 m diameter and weighs 7.6 kg as deployed with ballast chain. Operating on a solar-battery power system with five 2-W solar panels the Spotter buoy can be deployed indefinitely, although biofouling limits deployment duration in practice. The satellite receiver measures displacement using the Doppler shift and position data and records its displacement at 2.5 Hz, resulting in 4500 records per half-hour sampling period, which are stored on an SD data card. Spotter buoy versions 1 and 2 featured Iridium satellite telemetry of half-hourly onboard-computed wave spectra (coefficients) or a limited set of bulk wave parameters^42^ (describing wave height, period and direction), wind speed (inferred from measured wave spectra^43^) and buoy position. A water surface temperature sensor was added in version 2 and a cellular network data telemetry option in version 3.

To evaluate consistency in wave measurement between the Datawell and Spotter buoys we conducted a 2-month paired deployment at Old Bar (Fig. 1) during October-December 2018, where Datawell and Spotter buoys were deployed 100 m apart in similar water depths and on identical moorings. As both instruments measure displacement using the GNSS method, it was anticipated that the wave data measured by each should be comparable, although variations might emerge from different hull designs, satellite receiver units and firmware/software. The different displacement recording frequencies might also influence the derived wave data. The buoy comparison experiment found that wave data from the two instruments is comparable for our purposes, the key difference being in the occurrence and nature of data artefacts arising from satellite position observations. See the Technical Validation section for results of the Datawell-Spotter buoy comparison and further details on erroneous data from satellite position observations.

Deployments from March 2016 to December 2018 were made using a pool of four Datawell DWR-G4 buoys: two from NSW Environment and Heritage (NSWENV), one from the Sydney Institute of Marine Science (SIMS) through support from the Integrated Marine Observing System (IMOS), and one from the Water Research Laboratory (UNSW Sydney). Two Spotter-v1 buoys were purchased through SIMS-IMOS on behalf of NSWENV in June 2018. An additional four Spotter-v1 buoys were purchased by NSWENV in June 2019, three Spotter-v2 buoys in June 2021 and one Spotter-v3 buoy in April 2023. The Water Research Laboratory purchased a Spotter-v1 buoy in September 2019 to support ongoing deployments at Collaroy-Narrabeen Beach (Fig. 1). Following the Datawell-Spotter buoy comparison at Old Bar, the Datawell deployments (Table 1) at Farquhar and Old Bar (Fig. 1) were replaced with Spotter buoys (Table 2) during a service visit on 4 December 2018. Subsequently, Datawell buoys were only used at the Boomerang B deployment (Fig. 1), which was replaced with a Spotter buoy on 24 July 2019 (Table 2). The transition to Spotter buoys reduced the logistical requirements of maintaining deployments by increasing the duration between service visits, allowing for longer deployments and more simultaneous locations.

### Mooring designs and management

Our standard temporary mooring design follows Datawell’s recommendations for the DWR-G4 buoy in shallow water (p. 95)^41^ with some minor variations. They comprise a medium-size sand bottom boat anchor with at least 10 m of 8–10 mm galvanised steel chain to keep the mooring in place, and weighted Dyneema line connecting the anchor chain to the mooring floats and wave buoy at the water surface. This includes a length of line about twice the deployment water depth between the anchor chain and surface floats (e.g., 20–25 m of line for deployments around 12 m water depth). An additional 30 m length of unweighted line is added between the anchor chain and the weighted upper mooring line for deployments in 30 m water depth. Our weighted lines feature 3–4 elongate fishing sinkers (170 g) spliced and taped into the line about 1 m apart along the central section of line.

The surface floats for our standard mooring design include a pair of 0.25 m diameter spherical centre bore floats with 8.5 kg buoyancy rating each (Fig. 2a). In settings where increased mooring visibility is required (e.g., high vessel traffic), a 0.6 m base diameter yellow special navigation mark float (30 kg buoyancy rating) with a marine lantern is used with high-visibility surface line floats (Fig. 2b). The surface floats reduce the influence of the mooring on the movement of the wave buoy by maintaining slack line near the water surface between the floats and wave buoy. They also increase the visibility of the mooring to assist with navigation safety and reduce the chance of vessel strike. Both Datawell and Spotter wave buoys are equipped with yellow flashing navigation marker lights for night-time visibility.

For the standard mooring with paired surface floats (Fig. 2a), the wave buoy (with supplied ballast chain) is connected to the floats by 10–15 m of weighted line. For a high-visibility navigation mark mooring (Fig. 2b) the central sector of the weighted buoy line is replaced with a pair of 0.48 m length marker floats (9 kg buoyancy rating each) that are connected with 8 mm chain zip-tied along the float line grooves (Fig. 2b). Under the influence of changing currents or wind, the wave buoys can move freely within an arc (i.e., watch circle) around the mooring anchor point, while the surface floats absorb the motion of the mooring and reduce any influence on wave buoy displacement.

Moorings and buoys are susceptible to fouling by sessile marine fauna in the shallow coastal waters where our deployments are located, which left unchecked adds considerable weight to the mooring and reduces the efficacy of the surface floats in reducing mooring influence on the buoy. We have found that common marine fouling organisms in our settings include green “sea lettuce” algae (*Ulva sp*.), goose barnacles (*Lepas pectinata*), acorn barnacles (*Chthamalus antennatus*), sea squirts and sea tulips (*Pyura sp*.), and blue mussels (*Mytilus galloprovincialis*). Mooring entanglement on mobile marine fauna (e.g., whales and turtles) has not occurred with our buoy deployments to date, however, we are testing fauna-friendly mooring designs comprising flexible tubing sections around mooring lines to reduce any potential risk of marine fauna entanglement.

Datawell buoy deployments were visited every 4–5 weeks to change batteries and retrieve data, during which the buoy and mooring were cleaned onboard the vessel and redeployed, with the buoy and mooring replaced with fresh equipment every third visit. Spotter buoy deployments are visited every 4-6 months on average with both the buoy and mooring replaced with fresh equipment during each service visit. The dates of buoy deployments, service visits and buoy retrievals are provided in the readme metadata files with each data package. Heavy fouling that has been observed, upon visiting, to compromise wave buoy behaviour has only occurred when service visits were significantly delayed due to operational limitations (Fig. 2c,d). In such cases, data records have been inspected and truncated if they were deemed to be compromised – see Technical Validation (Data loss and omission).

Deployment, service and retrieval operations have been primarily carried out using our research vessels, *RV Bombora* (11.8 m fibreglass Stebercraft) and *Badoo* (6.4 m aluminium Seatamer). Deployment locations are recorded using RTK-GNSS survey or vessel navigation systems to ensure that moorings are redeployed at the same position during each service visit. The wave buoy and mooring lines are deployed first, and the chain and anchor are deployed last at the deployment location. Datawell buoys are switched on by connecting the power cable to the electronics unit several minutes prior to deployment. Spotter buoys are switched to Run (recording) mode moments prior to deployment and switched to Stand-by mode moments after retrieval. Further description of our deployment methods can be found within the Australian Wave Buoy Operations and Data Management Guidelines Manual^44^ available from Ocean Best Practices.

### Data processing

Our data processing methods follow long-established techniques used for spectral and time-domain analysis of surface buoy displacement measurements^45–47^. We apply our methods uniformly to the displacement data retrieved from onboard data cards in binary (Datawell) or ascii (Spotter) formats rather than using proprietary data processing tools. The key difference in the displacement data records between Datawell and Spotter buoys is that, due to the different sampling frequencies, in each half hour there are 2304 Datawell displacement records and 4500 Spotter displacement records. Both buoys commence measuring shortly (minutes) after they are powered-up (Datawell) or switched to Run mode (Spotter). By noting those times, and the times that they are deployed and retrieved from the water, we discard the beginning and end of the buoy displacement records and segment the data into regular half-hour packages beginning from the first UTC half hour passed following deployment.

We filter the raw displacement data to address known artefacts that affect GNSS wave buoy displacement measurements, which are caused by interruptions in the satellite data stream. The artefacts appear as erroneous spikes or sawtooth patterns in heave (*z*) displacements, in particular, which introduce errors in derived wave spectra and parameters^48–51^. Following an approach described by Andrews & Peach^51^, we apply a coarse spike filter to each half hour of displacement data to remove outliers exceeding five times the standard deviation and to improve the performance of high-pass filtering. We then apply a second-order Butterworth high-pass filter to remove any signals in the *x*, *y* and *z* displacement data that are beyond the recording frequencies of the buoys, using a cut-off of 0.029 Hz (Spotter buoy sampling limit). All subsequent processing is carried out using the filtered displacement set for each half-hour sampling period. Displacement data artefacts are further discussed in Technical Validation (Erroneous data).

For spectral analysis, the derivation of variance density spectra, spectral moments and bulk parameters follow similar standard approaches that are used by Datawell^41^ and Sofar Ocean^42^ to calculate onboard spectral and parametric data. Each continuous half-hour displacement record is divided into non-overlapping consecutive data segments (n = 256), from which consecutive spectra are derived and then averaged. Due to the varying sampling frequencies, each consecutive displacement data segment and derived spectra equates to sampling periods of 200 s (Datawell) and 102.4 s (Spotter). The wave spectra for each half hour from which bulk parameters are derived is therefore determined by averaging 9 (Datawell) or 17 (Spotter) consecutive spectra. The frequency resolution of the spectra also varies due to sampling rate, being 0.005 Hz for Datawell buoys^41^ and ≈ 0.0097 Hz for Spotter buoys^42^.

We use the fast Fourier-transform (FFT) method to derive surface elevation, *x*-displacement and *y*-displacement variance density spectra for each data segment, from which the *x*-*y* co-spectrum, *x*-*z* quad-spectrum and *y*-*z* quad-spectrum are constructed. Bulk parameters describing wave height and period for each half-hour displacement data record are calculated from the frequency moments of the variance density spectra as described in Table 4. Directional properties are estimated from the lowest-order directional moments using the co/quad-spectra and displacement spectra^45,46^. Directions associated with the mean and peak frequencies are calculated as described in Table 4.

**Table 4:** Metadata fields, spectral and time-domain wave parameter fields, and data quality fields, shown in the order that they appear in the time series wave parameter CSV data tables. Corresponding names for netCDF data fields are shown in square brackets.

Notation	Parameter	Description	Units
—	Serial	Serial number of the wave buoy that recorded the data (DW: Datawell buoy; SPOT: Spotter buoy)	—
—	Time [TIME]	Date-time (AEST) at the end of the half-hour observation period from which the wave parameters were derived	—
—	Lat [LATITUDE]	Latitude of the wave buoy at the end of the half-hour observation period	°
—	Lon [LONGITUDE]	Longitude of the wave buoy at the end of the half-hour observation period	°
—	Temp [TEMP]	Surface water temperature (only available for buoys with temperature sensor)	° C
*H*_*m*0_	Significant wave height [WSSH]	Four times the square root of the zeroth-order moment of the non-directional wave spectrum: 4∙$\sqrt{{m}_{0}}$	m
*H*_*sig*_	Significant wave height [WHTH]	Average of the top 1/3 of trough-crest wave heights identified using the zero-upcrossing analysis method	m
*H*_*max*_	Maximum wave height [WMXH]	The highest trough-crest wave height identified using the zero-upcrossing analysis method	m
*T*_*z*_	Mean wave period [WPMH]	Average wave period determined using the zero-upcrossing analysis method	s
*T*_*m*02_	Mean absolute wave period [WPSM]	Square root of ratio of the zeroth and second-order moments of the non-directional wave spectrum: $\sqrt{{m}_{0}/{m}_{2}}$	s
*T*_*m*01_	Mean wave period [WPFM]	Ratio of the zeroth and first-order moments of the non-directional wave spectrum: ${m}_{0}/{m}_{1}$	s
*T*_*P*_	Peak wave period [WPPE]	Period corresponding to the discrete frequency of the non-directional spectra with maximum energy density	s
*θ*_*m*_	Mean wave direction [SSWMD]	Mean wave direction derived from the lowest order bulk directional moments (TN: true north)	° TN
*θ*_*p*_	Peak wave direction [WPDI]	Wave direction from directional moments corresponding to the discrete frequency with maximum energy density (TN: true north)	° TN
*σ*_*m*_	Mean wave direction spreading [WMDS]	Directional spreading around the mean wave direction	°
*σ*_*p*_	Peak wave direction spreading [WPDS]	Directional spreading around the peak wave direction	°
—	Qflag [WAVE_quality_control]	Primary quality control flag that grades data as pass (1), suspect (3), fail (4) or absent (9), see Table 5	—
—	Qcode	Secondary quality control code that describes the provenance, completeness and quality of the time series wave parameter data, see Table 5	—
—	Percent	Proportion of the full half hour ending at the timestamp that displacement data were successfully recorded	%
—	Dof	Degrees of freedom for spectral averaging	—

We use standard zero-upcrossing analysis methods^47^ to calculate time-domain wave height and period parameters using the same regular half-hour buoy displacement data packages that are used for spectral processing. Time-domain parameters are computed for comparison with corresponding spectral parameters and to provide a comprehensive and flexible suite of parameters to support most applications. The time-domain parameters are described in Table 4. The time series data table of processed spectral and time-domain wave parameter data is constructed by appending the parameter values calculated from each consecutive half-hour buoy displacement record.

A key difference between the processed data from completed deployments and the data computed onboard and transmitted from Spotter buoys in real time (which is provided for active deployments, see Data Records) is that our processed data are derived from half-hour displacement data packages commencing on the hour and half hour. In contrast, real-time data that is computed onboard and transmitted from Spotter buoys derive from half-hour displacement data segments that commence from whatever time the buoy was switched to Run mode and began sampling. This is important for using the nearshore wave buoy data with concurrent data from wave hindcast/forecast models (in regular UTC time) or the offshore wave buoy network along the NSW coast^13^ (Fig. 1). The offshore buoys are large-format (0.9 m hull diameter) Datawell Waverider buoys that synchronise data capture and processing to regular UTC hours, with both real-time and post-processed data provided in Australian Eastern Standard Time (AEST)^13^. Following Datawell^41^ and Sofar Ocean^42^ conventions for onboard processed buoy data, our wave parameter time-series data are time-stamped with the end time (AEST) of each half-hour displacement dataset from which the parameters were derived.

### Quality control

Our quality control objectives are to preserve the maximum amount of data measured by the wave buoys, while identifying for the end user, any data that may be compromised or less rigorous and which therefore should be interpreted and used with caution. We follow published standards for oceanographic data quality control and flagging^52–54^ with some modifications and extensions as described below. In our data records, we prioritise post-processed wave parameter data derived following our methods above (see Data processing), but also provide onboard computed wave parameter data for any timestamps where buoy displacement data were not recovered.

Four data quality fields (*Qflag*, *Qcode*, *Percent*, *DoF*; Table 4) are included in our parameter time-series data tables to describe the rigour and perceived quality of each data point.

The *Percent* field shows the proportion of each half-hour period that displacement data were recorded by the buoy. A value of 100 indicates that a full half-hour set of displacement data (e.g., 2304 records for a Datawell, 4500 records for a Spotter) was recorded and used in the data processing. Values between 0 and 100% indicate partial loss of displacement data, which may occur due to satellite receiver obstruction, data card write issues or during a mooring service when there is no instrument in the water. A *Percent* value of zero indicates that no displacement data were recorded during that half hour sampling period. The *DoF* field provides the corresponding degrees of freedom for the statistical calculation of wave spectra from which spectral parameters are derived. Due to different sampling rates, the degrees of freedom corresponding to a complete displacement data record (*Percent* = 100) are 36 for a Datawell buoy and 68 for a Spotter buoy. See Technical Validation (Data loss and omission) for more information on data completeness.

The *Qflag* field provides a primary level quality flag consistent with published standards^52–54^ that identifies each data point as pass (1), suspect (3), fail (4) or absent (9) based on the results of diagnostic testing. Users wishing to exclude suspect, erroneous or absent data points from their analysis or applications can use the *Qflag* field to sort, extract or omit data without further interrogation. Of the deployment data summarised in Tables 1, 2, over 97% of half-hourly data points were evaluated as pass by diagnostic tests (Table 5).

**Table 5:** Primary quality flag (*Qflag*) and secondary quality code (*Qcode*) values that describe the provenance, completeness and quality of the wave parameter data provided in the time-series data tables (Table 4).

Qflag	Qcode	Description of wave buoy data provided	Percent (*p*) value	% occurrence
1	1	Parameters calculated using **displacement data, complete** buoy displacement record on data card, data **passes** quality control tests	100	97.517
3	2	Parameters calculated using **displacement data, incomplete** buoy displacement record on data card, data **passes** quality control tests	23 ≤ *p* < 100	0.022
3	3	Parameters from r**eal-time data** record are provided, **limited or absent** buoy displacement record on data card, data **passes** quality control tests	0 ≤ *p* < 23	0.020
3	4	Parameters calculated using **displacement data, sufficient** buoy displacement record on data card, quality control tests identify data as **suspect**	23 ≤ *p* ≤ 100	1.101
3	5	Parameters from **real-time data** record are provided, **limited or absent** buoy displacement record on data card, quality control tests identify data as **suspect**	0 ≤ *p* < 23	0
4	6	Parameters calculated using **displacement data, complete** buoy displacement record on data card, data **fails** quality control tests	100	0.549
4	7	Parameters calculated using **displacement data, incomplete** buoy displacement record on data card, data **fails** quality control tests	23 ≤ *p* < 100	0.001
4	8	Parameters from **real-time data** record are provided, **limited or absent** buoy displacement record on data card, data **fails** quality control tests	0 ≤ *p* < 23	0
9	9	**No data** available – e.g., buoy service visit, batteries exhausted, instrument failure.	0	0.789

Corresponding *Percent* values (Table 4) and the proportion of all half-hourly data records (n = 323,584) for which each *Qcode* value occurred for all completed deployments (Tables 1–2) are also provided.

Suspect data (*Qflag* 3) are identified using range and rate of change tests^53^, with a data point flagged as suspect if any parameter value violates the test condition for that parameter. Range tests flag parameter values outside of acceptable ranges as follows: *H*_*m0*_ (0.1–10 m), *T*_*p*_ (3–21 s), *σ*_*p*_ (0.07–80°) and temperature (5–55°). Rate of change tests flag change in parameter values between successive half-hour timesteps that exceed acceptable limits as follows: *H*_*m0*_ (2 m), *T*_*p*_ (10 s), *θ*_*p*_ (50°), *σ*_*p*_ (25°) and temperature (2°).

Anomalous data that are likely compromised by displacement measurement errors (*Qflag* 4) are identified using a mean and standard deviation filter^53^, which is applied to *H*_*m0*_ and *T*_*m02*_ (*T*_*m01*_ for real-time Spotter data as *T*_*m02*_ is not provided) using a 6 hour (n = 12) moving window and 2-standard deviation filter. A data point is flagged as fail (*Qflag* 4) if both the wave height and period parameter values within the moving window exceed their respective filter values. All data points where *T*_*p*_ > 21 s are also flagged as fail. The tests capture wave parameter values derived from erroneous spectra that arise due to displacement data artefacts from satellite receiver errors^48–51^. See Technical Validation (Erroneous data) for further details.

The *Qcode* field provides a secondary level quality code that summarises the provenance, completeness and quality of the data provided for each half hour period. Table 5 lists all potential *Qcode* values, including their relation to *Qflag* values, and describes the nature of the displacement data recorded by the buoy and the wave parameter data provided in the time series table for each case. The rate of occurrence of each *Qcode* value is also provided.

Data with *Qcode* values 1–3 passed diagnostic tests, however, for 2 an incomplete but sufficient buoy displacement data record was available for data processing, while for 3 an absent or insufficient (*Percent* < 23) displacement data record was present and onboard computed (real-time) wave parameter data are provided instead. The threshold of 23% displacement data retention for each half-hour timestamp, which is the minimum used to calculate parameters from displacement data stored on the buoy data card, equates to a minimum of 4 Spotter spectra for spectral averaging (*DoF* = 16). The Datawell buoys either store a complete displacement record or no displacement data for each half-hour sampling period. When *Percent* < 23 (including *Percent* = 0), wave parameter values (if available) processed onboard Spotter buoys for the irregular timestamp falling within the regular half-hour sampling period are included in the parameter time series data instead (*Qcode* 3, 5 and 8, Table 5). This is because in some cases, parameter data may have been computed and transmitted by the buoy within a half-hour sampling period during which few or no displacement data were recorded to the data card. A *Qflag* value of 3 (suspect) is applied when data pass diagnostic tests but *Percent* < 100.

A *Qcode* value of 4 or 5 indicates that some parameters at that timestamp were flagged as suspect by diagnostic tests (*Qflag* 3), whether data provided were calculated from buoy displacements following our methods (*Qcode* 4), or onboard computed parameters have been provided (*Qcode* 5). *Qcode* values 6, 7 and 8 indicate that some parameters at that timestamp failed diagnostic tests (*Qflag* 4), and the parameters provided were calculated from either a complete (*Qcode* 6) or incomplete (*Qcode* 7) displacement record, or onboard computed parameters have been provided (*Qcode* 8). *Qcode* value 9 always occurs with *Qflag* value 9, following standard conventions for identifying missing data^52–54^, and indicates that neither displacements nor real-time data were available for that timestamp (e.g., buoy failure or a mooring service visit).

All half-hour timestamps from buoy deployment to retrieval are included in the time series data tables regardless of missing data points (see Data loss and omission), which can be readily identified by the *Qflag*/*Qcode* values. An exception is if the data record has been truncated at the end of a deployment to omit data that were either deemed to be compromised by mooring influence from biofouling or were absent due to buoy failure. The number of occurrences of each *Qflag*/*Qcode* combination in each completed deployment data record is provided in the readme file included with each data package, and the proportion of each *Qcode* in the dataset (all deployments) to date are provided in Table 5. It is at the user’s discretion to include or exclude data in their application based on their needs and appraisal of data quality.

Readers are directed to Technical Validation for further details on: Offshore-nearshore buoy comparison, Datawell-Spotter buoy comparison, Erroneous data, and Data loss and omission.

## Data Records

A static version of the wave buoy displacement data, spectral data and wave parameter time series data in netCDF format, for all completed deployments at the time of publication (Tables 1, 2), is available from the Australian Ocean Data Network^55^ to accompany this data descriptor. The static dataset will not be updated as underway (Table 3) and future wave buoy deployments are completed.

Many data users will be interested in accessing the wave parameter data in CSV format. The NSW Sharing and Enabling Environmental Data (SEED) portal^56^ hosts an up-to-date version of the wave parameter time series data (completed deployments)^57^ in CSV format within zip packages that also contain data summary plots and tables (described below). The SEED portal also includes an online viewer for real-time wave parameter time series data (active deployments)^58^ from underway wave buoy deployments. New data packages will be added to the SEED portal upon the completion of underway and future wave buoy deployments.

For advanced data users, up-to-date versions of the wave buoy displacement data, spectral data and wave parameter time series data in netCDF format are available from the Australian National Wave Archive dataset on the Australian Ocean Data Network^59^. The netCDF data formats included in the static and National Wave Archive datasets are described further below.

Readers are directed to the Usage Notes for details on complementary data that may be useful for data applications, including high-resolution coastal bathymetry and landforms data covering the entire NSW coastline and offshore (deep-water) wave buoy data (Fig. 1).

The nearshore wave buoy datasets described here contribute to the Australian Research Data Commons (ARDC) project, *Catching Oz Waves: National infrastructure for in-situ wave observations*^60^.

### Wave parameter time series data (completed deployments) in CSV format

Data packages as described here for completed wave buoy deployments can be downloaded from the SEED portal dataset page^57^ under *Dataset Packages*.

Each deployment data package comprises one zip folder containing the parameter time series data table and wave height-direction exceedance tables in CSV format, summary plots of the deployment data, and a readme file containing metadata about the entire deployment and individual service deployments. The naming format for the data packages is as follows and is also included as a prefix for all files within each data package:

$${\bf{NSWENV}}\_{\bf{StartDate}}-{\bf{EndDate}}\_{\bf{WaterDepth}}\_{\bf{Location}}\_{\bf{WAVE}}\_\left[{\bf{FILE}}\right]$$

**NSWENV**: NSW Environment Department

**StartDate**: start of deployment date in YYYYMMDD

**EndDate**: end of deployment date in YYYYMMDD

**WaterDepth**: water depth below sea level (Australian Height Datum - AHD) to the nearest metre (m)

**Location**: name of deployment location (Tables 1–3)

**WAVE**: wave data type

Three data tables (CSV format) are included in each deployment data package:

1. A parameter time series data table [**Parameters**] containing a set of widely used spectral and time-domain wave parameters with metadata and data quality fields (Table 4). Data are provided at half-hour temporal resolution for the duration of each deployment and are time-stamped (AEST) with the end time of the half-hour buoy displacement record from which they were derived.
2. Two wave height-direction exceedance tables based on the mean (θ_*m*_) [**ExceedanceDm**] and peak (θ_*p*_) [**ExceedanceDp**] wave directions as summarised in the provided wave rose plots (see below). Fail data points (Table 5) are omitted from the exceedance table calculations.

Data plots (PNG format) are included with each deployment data package to allow users to quickly inspect and interpret the data measured during each deployment:

1. A summary time-series plot [**Summary**] showing key wave height (*H*_*m0*_, *H*_*max*_), period (*T*_*m02*_, *T*_*p*_,) and direction (θ_*m*_, θ_*p*_) parameters over the duration of each deployment, which also identifies data points that were flagged as suspect or fail by diagnostic tests (Fig. 3).

**Fig. 3:**
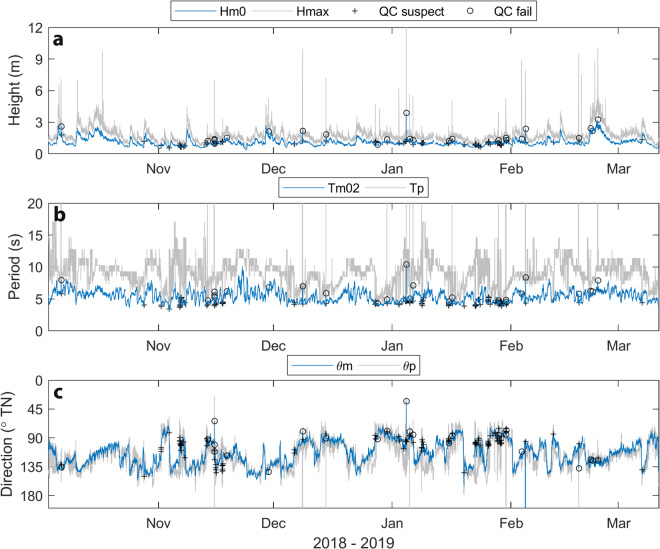
Example time series parameter data summary plot from Spotter buoy deployments at Old Bar B (Fig. 1, Table 2) showing measured wave height (**a**), period (**b**) and direction (**c**) parameters. Marker symbols on the mean parameter (*H*_*m0*_, *T*_*m02*_ and θ_*m*_) time series in all plots identify 126 (from a total of 7,615) data points that were flagged as suspect (+) and 25 data points that failed (o) quality control tests (Table 5).
2. A wave power time-series plot [**Power**] showing the total incident and alongshore wave power over the duration of each deployment, where alongshore power is relative to the average orientation of the shoreline adjacent to the deployment, which is provided in each deployment readme file.
3. Two wave rose plots based on the mean (θ_*m*_) [**RoseDm**] and peak (θ_*p*_) [**RoseDp**] wave directions. Fail data points (Table 5) are omitted from the wave rose plots.
4. A sea surface temperature plot [**SeaTemp**] showing water temperature measured by the wave buoy (only included if the instruments deployed had temperature sensors).

A readme file [**Readme**] (TXT format) is also provided and contains additional details about individual buoy service deployments and the entire deployment set for that location. Buoy deployment and retrieval dates, first and last data (recorded) dates, and mooring centroids and water depths (from high-resolution seabed mapping, see Usage Notes) are provided for each service deployment and for the entire deployment set, along with the total number of *Qflag*/*Qcode* value occurrences in the entire deployment dataset (Table 5).

A shapefile of buoy deployment locations for use in Geographical Information Systems (GIS) is also provided on the SEED portal dataset page^57^ and will be updated as new deployment data packages are added.

### Wave parameter time series data (active deployments) via online viewer

Real-time data received via satellite transmission from active wave buoy deployments can be viewed and downloaded from the SEED portal dataset page^58^ (see instructions below).

A rolling 7-day window of key wave parameters^42^ (*H*_*m0*_, *T*_*m01*_, *T*_*p*_, θ_*m*_, θ_*p*_; Table 4), inferred wind speed and direction estimated from the wave spectra^43^, and surface water temperature (Spotter-v2/v3 deployments only) are provided for each active deployment in charts and data tables at half-hour intervals. The data are computed onboard the buoys following the manufacturers methods and are transmitted via satellite or cellular telemetry. They are not quality assessed or controlled in any way. Various factors may cause erroneous data points and users are advised to exercise caution when using the data. Real-time data are provided for information purposes and should not be solely relied upon for coastal hazard advice or to guide operational activities.

Select *Map Viewer* from the SEED portal dataset^58^ to open a Geocortex SEED Map and browse active wave buoy deployment locations, view real-time wave parameter time series data plots and tables, and download tabulated time series data. Real-time chart and table data can be browsed by clicking on a buoy location marker, selecting ‘View additional details’ in the data summary pop-up window, and then selecting ‘Show Expanded View’ from the drop-down menu list to change the side bar to landscape view. Data plots can be browsed using the ‘Charts’ tab and tabulated data using the buoy data type tabs.

### Displacement, wave spectra and parameter data (advanced users) in netCDF format

Advanced users interested in wave buoy displacement data, wave spectra data and parameter time series data from the NSW nearshore wave buoy program can access the data in netCDF format via the THREDDS catalog on the Australian National Wave Archive^59^. Open the ‘NetCDF files via THREDDS catalog (NSW-DPE)’ link on the National Wave Archive dataset page to browse netCDF data by type and deployment location. The netCDF data are in the same format as the static dataset^55^ but will be updated upon the completion of underway (Table 3) and future deployments.

The WAVE_RAW_DISPLACEMENTS (buoy displacement) netCDF data fields include: TIME (displacement measurement time), TIME_LOCATION (location measurement time), LATITUDE (WGS84), LONGITUDE (WGS84), XDIS (east displacement), YDIS (north displacement) and ZDIS (heave displacement).

The WAVE_SPECTRA (spectral data) netCDF data fields include: TIME (half-hourly measurement interval), FREQUENCY (measurement frequency), LATITUDE (WGS84), LONGITUDE (WGS84), A1 (first term of Fourier cosine series), A2 (second term of Fourier cosine series), B1 (first term of Fourier sine series), B2 (second term of Fourier sine series) and ENERGY (energy density).

The WAVE_PARAMETERS (parameter data) netCDF data fields are indicated in square brackets in Table 4. All time fields in the netCDF data are expressed in days since 1/1/1950 00:00:00 UTC.

## Technical Validation

### Sampling approach

The accuracy of *in-situ* wave measurements using moored buoys, including GNSS buoys, has been investigated in laboratory and field studies and reported elsewhere^61–65^. The wave buoys used in this program measure their displacement using satellite positioning receivers and do not require calibration following manufacture as they do not feature any mechanical sensors. While the influences of the mooring on buoy motion cannot be completely avoided in all conditions, our moorings are fit for purpose and follow the manufacturer’s recommendations for shallow deployments (p. 95)^41^. The nearshore locations of our deployments are not typically subject to strong and sustained currents that may influence mooring behaviour at offshore deployment sites. Our data processing methods for spectral and time-domain derivation of wave parameters from surface buoy displacement data follow well-established techniques^45–47^ that are used by the buoy manufacturers for onboard data processing^41,42^.

Biofouling is a pervasive factor that is known to influence mooring behaviour and buoy motion when the fouling compromises the buoyancy properties of the mooring, which may lead to damping of high frequencies and/or introduce artefacts at low frequencies due to increased drag^66–69^. We observe mooring behaviour and buoy motion upon arriving at each deployment during service visits and retrievals to assess if the mooring might be compromised and inspect fouling upon removing equipment from the water. On the few occasions when heavy fouling due to a delayed service visit has been assessed as potentially impairing buoy motion (e.g., Fig. 2c,d), the processed data has been compared with concurrent data from the nearest offshore wave buoy (Fig. 1) and scrutinised for any changes in parameter behaviour (e.g., damping) through the deployment period. To date, this has resulted in truncation of affected datasets from the Collaroy 3 and Merimbula deployments (see Data loss and omission). Data from a heavily fouled deployment at Boomerang B shown in Fig. 2c was not affected as the batteries in the Datawell buoy had run flat prior to heavy fouling.

### Offshore-nearshore buoy comparison

We compared wave data measured by nearshore wave buoys at Stockton, Figure Eight Pools and Broulee with concurrent deep-water wave data from the nearest offshore wave buoys at Sydney, Wollongong and Batemans Bay respectively (Fig. 1). Those nearshore deployment locations each had more than one year of observations and cover a range of settings, including: an embayed southern corner in 13 m water depth (Stockton), an open coast in 30 m water depth (Figure Eight Pools), and an embayed northern corner in 13 m water depth (Broulee).

**Fig. 4:**
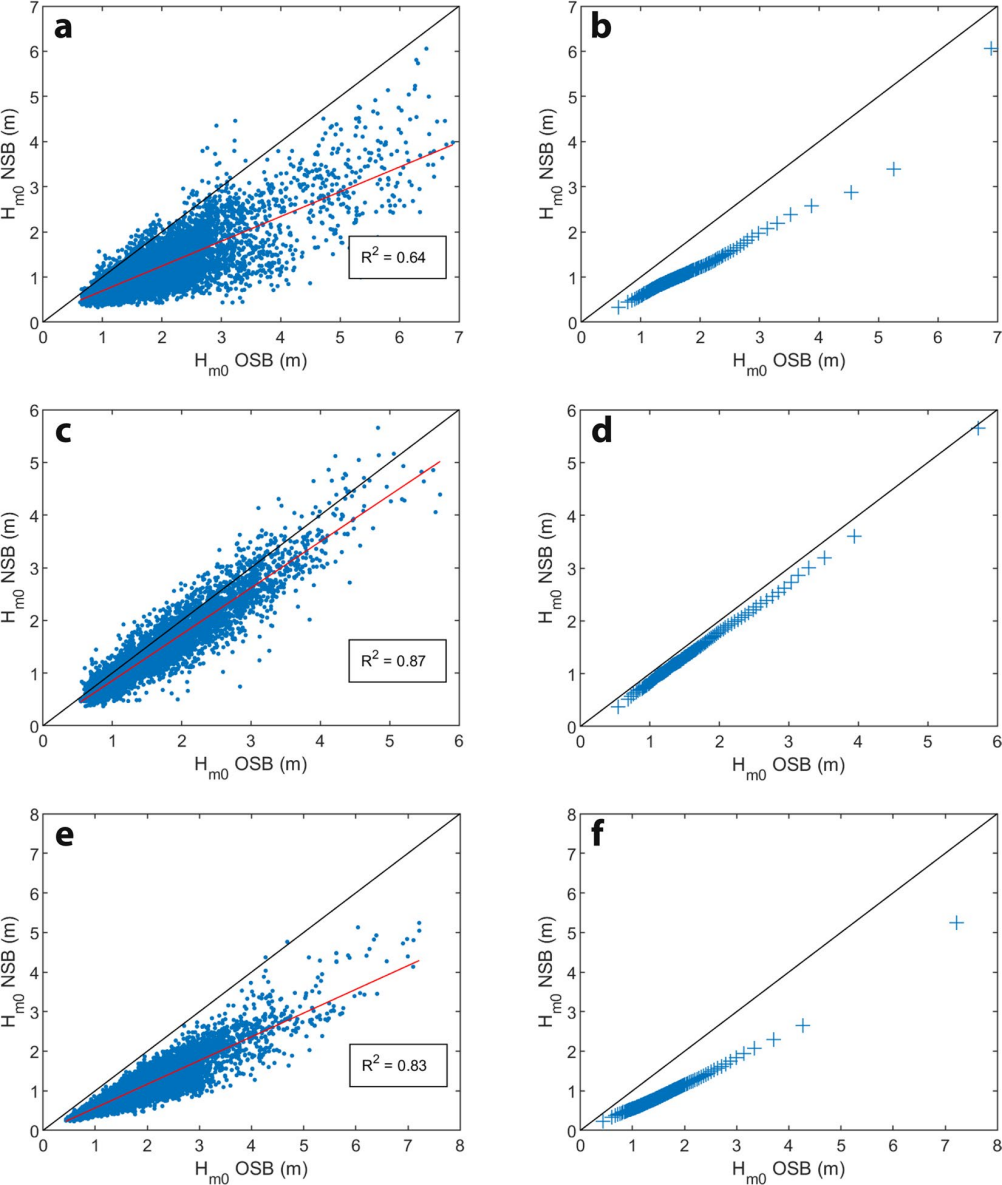
Scatter plots (left) and quantile-quantile plots (right) comparing significant wave height (H_m0_) measured concurrently by nearshore wave buoys (NSB) and offshore wave buoys (OSB) at: (**a,b**) Stockton (13 m depth) and Sydney (90 m depth); (**c,d**) Figure Eight Pools (30 m depth) and Wollongong (80 m depth); and, (**e,f**) Broulee (13 m depth) and Batemans Bay (65 m depth). See Fig. 1 for NSB and OSB locations and Tables 1,2 for NSB deployment details.

Scatter and quantile-quantile plot comparisons of *H*_*m0*_ between the nearshore-offshore wave buoy pairs capture the varying influence of wave transformation processes on wave height attenuation from deep to shallow water between sites (Fig. 4). In all cases, wave heights are reduced from deep water (offshore buoy) to shallow water (nearshore buoy), consistent with expected wave attenuation. The greater scatter for the embayed Stockton and Broulee sites relative to Figure Eight Pools suggests higher sensitivity of wave height attenuation to other characteristics of the wave field (e.g., wave period and direction), in embayed settings. The lower wave height attenuation at Figure Eight Pools is consistent with the steep bathymetry and open coastline there, and the 30 m deployment water depth at that site (Fig. 1).

The offshore-nearshore wave height comparisons demonstrate that the nearshore wave buoy deployments are capturing patterns of deep- to shallow-water wave transformation as would be expected from consideration of the deployment settings and emphasise the importance of the nearshore wave buoy data for understanding local wave climates for coastal research and management applications.

### Datawell-Spotter buoy comparison

Deployments at our first eight locations were commenced and completed using Datawell buoys (Table 1). During the concurrent deployments at Farquhar and Old Bar in 2018 (Fig. 1), we carried out a direct comparison between the Datawell DWR-G4 buoy at Old Bar and two new Spotter-v1 buoys, to assess whether the wave data measured by the two instruments could be considered equivalent for our purposes. A Spotter buoy was deployed at Old Bar B, 100 m to the south of the Datawell buoy at Old Bar A in similar water depth (Table 2). The paired Old Bar deployments were maintained for two months (3 October-4 December 2018), with a service visit on 23 October to change the Datawell buoy batteries and swap the Spotter buoy (to test both new Spotters). At the end of the comparison, the Datawell buoy at Old Bar A was removed (Table 1) and the Spotter deployment was maintained at Old Bar B until 11 March 2019 (Table 2). We processed the displacement data collected by the adjacent Datawell and Spotter buoys using uniform methods as previously described, see Methods, (Data processing).

**Fig. 5:**
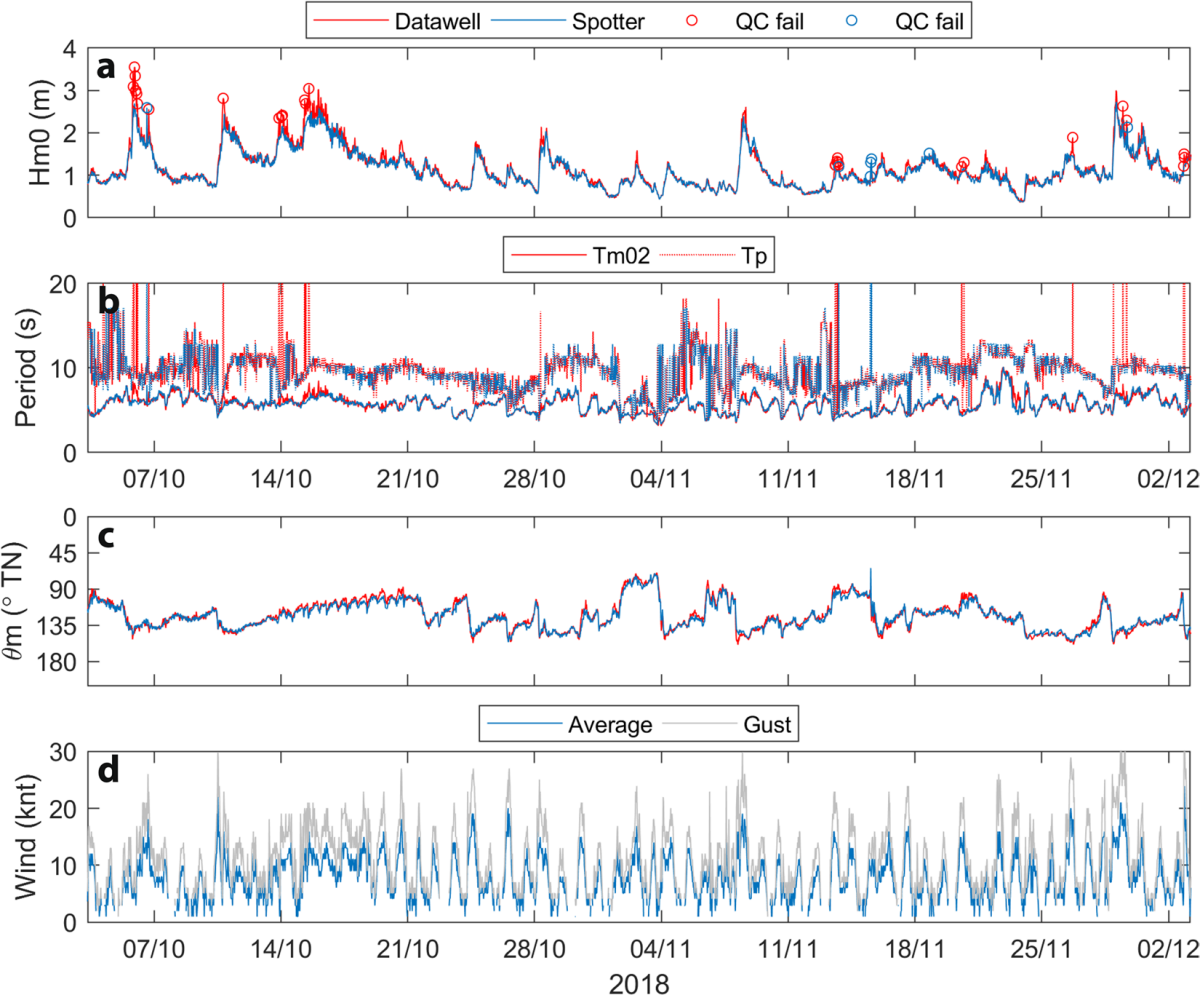
Measured wave height (**a**), period (**b**) and direction (**c**) data from the 2-month Datawell-Spotter buoy comparison at Old Bar (Fig. 1c), where Spotter buoys were deployed at Old Bar B (Table 2), 100 m south of the concurrent Datawell deployment at Old Bar A (Table 1). Circle markers on the *H*_*m0*_ time series identify Datawell (red) and Spotter (blue) data points that failed quality control tests (*Qflag* 4, Table 5). Also shown are (**d**) wind speeds (average and gust) measured by anemometer at Port Macquarie airport (66 km north of Old Bar) during the deployments.

The time series of concurrent wave data over the 2-month comparison period indicates close agreement between key wave height, period and direction parameters measured by the two instruments, showing consistency for most conditions and some notable deviations (Fig. 5). The Datawell buoy tended to predict higher significant wave height (*H*_*m0*_) during energetic conditions, which were often accompanied by spikes in peak period (*T*_*p*_). Such data points (red circles in Fig. 5) typically failed diagnostic tests (*Qflag* 4) and emerge from artefacts in the displacement data that are further discussed below (see Erroneous data). Mean wave period (*T*_*m02*_) and mean wave direction (θ_*m*_) compared well with minor deviation on some occasions when *H*_*m0*_ measured by the Datawell buoy crept above that measured by the Spotter buoy. Wave roses generated from the Datawell and Spotter data (not shown here) were very consistent.

**Fig. 6:**
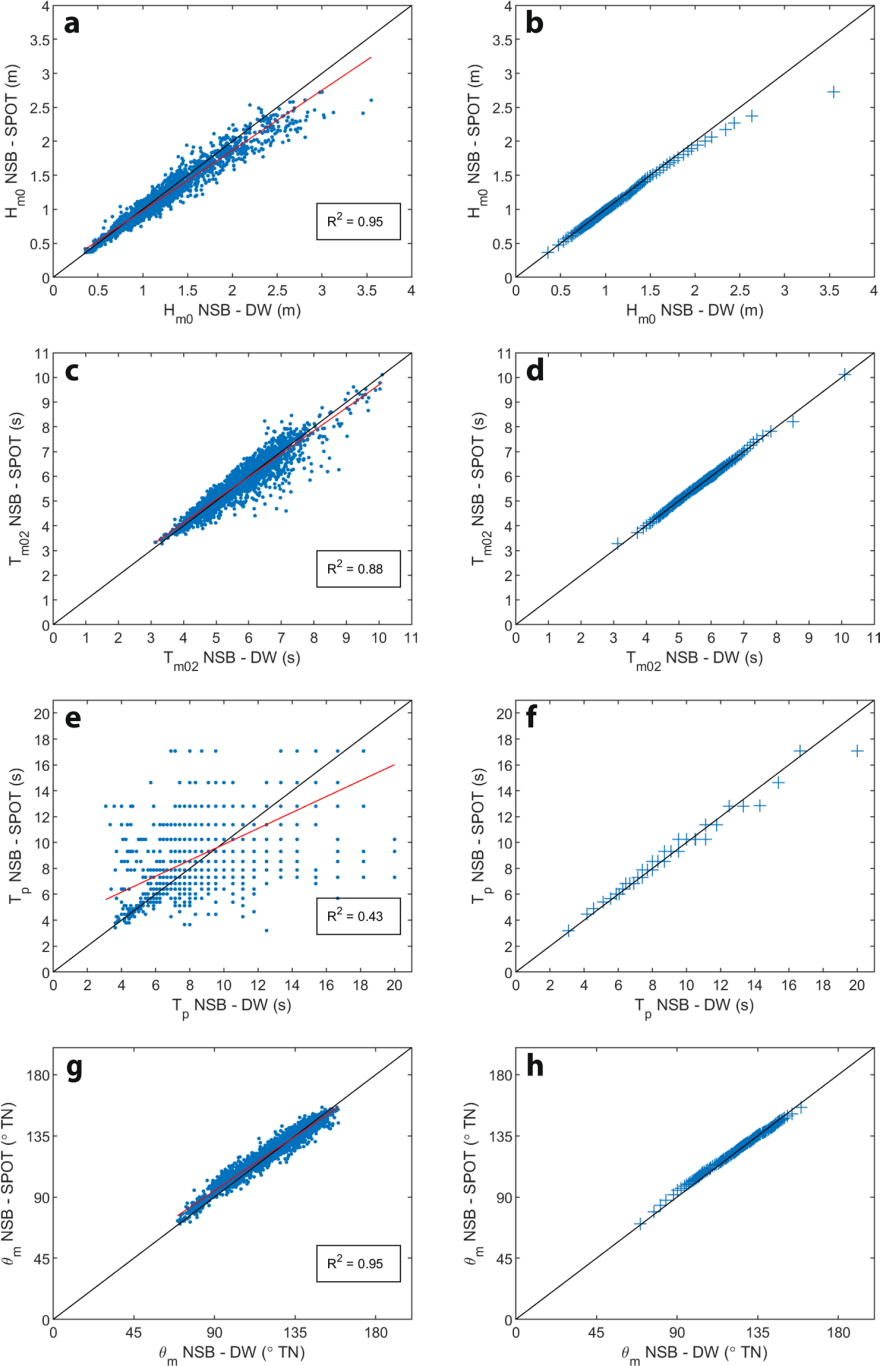
Scatter plots (left) and quantile-quantile plots (right) comparing significant wave height (H_m0_; **a,b**), mean wave period (T_m02_; **c,d**), peak wave period (T_p_; **e,f**) and mean wave direction (θ_m_; **g,h**), measured by Datawell (DW) and Spotter (SPOT) wave buoys located 100 m apart at Old Bar A and Old Bar B respectively (Fig. 1c), during the 2-month comparison deployments (Fig. 5). Data pairs for timestamps at which Datawell or Spotter data failed quality control tests (marked by red and blue circles respectively in Fig. 5) were omitted from the data comparison plots. Data comparison statistics are provided in Table 6.

Scatter and quantile-quantile plots provide a more informative comparison of wave data recorded by the Datawell and Spotter buoys (Fig. 6). Buoy data pairs for timestamps where either Datawell or Spotter data failed quality control tests (marked by circles in Fig. 5) were omitted from the statistical comparisons. The positive bias in *H*_*m0*_ for the Datawell buoy is evident for larger wave heights, however, inspection of Fig. 5 shows that for instances of *H*_*m0*_ > 2 m when data passed quality control tests, measured wave heights were similar. In comparison, *T*_*m02*_ and θ_*m*_ compare very well across the observed ranges. The positive bias in Datawell peak periods for *T*_*p*_ > 14 s derives from the varying frequency resolution between the two instruments, which is evident in the *T*_*p*_ scatter plot. Oscillation in spectral peak parameters for conditions with multiple energy spectrum peaks makes comparisons of *T*_*p*_ only generally informative. Table 6 provides standard comparison statistics describing the closeness of fit between wave parameters measured by the Datawell and Spotter buoys at Old Bar and shows similar consistency for spectral and time-domain parameters.

**Table 6:** Comparison statistics for data measured by Datawell and Spotter buoys (Fig. 6) during the 2-month concurrent deployments at Old Bar A (Table 1) and Old Bar B (Table 2) respectively.

	R2	RMSE	Bias	Scatter Index	Symmetric Slope	Skill Score
*H*_*m*0_	0.947	0.116	−0.037	0.100	0.885	0.982
*H*_*sig*_	0.972	0.069	−0.009	0.065	0.974	0.993
*H*_*max*_	0.759	0.434	−0.082	0.231	0.728	0.920
*T*_*z*_	0.888	0.385	−0.173	0.064	0.929	0.963
*T*_*m*02_	0.883	0.344	0.004	0.060	0.930	0.969
*T*_*m*01_	0.834	0.474	0.008	0.073	0.881	0.954
*T*_*P*_	0.432	1.805	0.021	0.189	0.616	0.792
*θ*_*m*_	0.953	4.230	1.055	0.035	0.890	0.985
*θ*_*p*_	0.630	12.317	0.877	0.099	0.737	0.883

Data pairs for timestamps at which Datawell or Spotter data failed quality control tests (i.e., marked by red and blue circles respectively in Fig. 5) were omitted from the data comparison statistics.

Considering the complex bathymetry at Old Bar (Fig. 1), minor variations in wave parameters measured by the Datawell and Spotter buoys might be expected due to random wave fields and fine-scale variability in wave transformation in that setting. The closeness of fit between the two datasets thus demonstrates that the Spotter buoy provides comparable *in-situ* wave observations to the Datawell buoy, and the two measurement platforms can be considered equivalent for the purposes of our program. The results of our buoy comparison experiment are consistent with those previously reported that document strong agreement between parametric wave data from measurements made by Datawell DWR-G and Spotter GNSS buoys, and also motion sensor buoys (e.g., Datawell DWR-MkIII and DWR-4, TriAXYS)^51,70–73^. The key difference between data collected using Datawell DWR-G4 and Spotter buoys in our program appears to be in the occurrence and nature of artefacts in the recorded displacement data that lead to erroneous wave spectra and derived wave parameters.

### Erroneous data

A weakness of GNSS wave buoys that use a satellite receiver to measure their displacement, rather than internal motion sensors, is that measurements can be affected when the satellite receiver is obstructed, for example by saltwater overtopping during collision of unbroken or breaking sea waves, or simply if insufficient satellites are in range to accurately measure buoy displacement using Doppler shift or position data^48,51,61,63^. For this reason, the Datawell DWR-G4 buoy is only rated for moored deployments in currents less than 1 m/s, as the “bow wave” of the buoy under drag could overtop the hull^41^. In Datawell GPS buoy displacement data, an artefact caused by satellite data interruption manifests as a sawtooth pattern and introduces erroneous low-frequency energy into derived wave spectra^48–51^. The artefact is not attributed to the mooring influence (although that might exacerbate it) but derives from temporary disruption to the satellite data stream. The Datawell satellite receiver accesses a smaller pool of GPS satellites than Spotter GNSS buoys, which operate on the Iridium satellite network.

We compared wind data from an anemometer at Port Macquarie Airport (66 km north from Old Bar) with measured wave data during the Old Bar buoy comparison experiment (Fig. 5) to investigate if fail data points (*Qflag* 4) deriving from Datawell and Spotter displacement data artefacts correlated with above average winds. Above-average wind speeds were experienced prior to and/or during most of the 24 and 7 instances of fail data (*Qcode* 5) in the Datawell and Spotter records respectively. A complete displacement record (*Percent* = 100) was stored on the data cards for all instances of fail data, and thus the erroneous data points were caused by artefacts in the displacement data rather than insufficient displacement data. For notable occurrences of high wind speeds but no fail wave data points, wind directions were found to play a role. For example, wind speeds exceeded 18 knots with gusts to 30 knots on 8/11/2018, however, wind directions were from the south-west (230–250° TN), meaning there was minimal fetch at Old Bar between the shore and the wave buoys (Fig. 1). While only a qualitative analysis, the comparison between wave buoy data and coastal wind observations suggests that the displacement artefacts experienced by both buoys may occur during above-average winds, and thus are likely caused by saltwater overtopping of buoys during short-sea conditions.

Strong winds and choppy seas do not always compromise the performance of GNSS buoys, however. For example, during 16–17 July 2021, strong WNW winds generated choppy sea at Deeban over a 2 km fetch (Fig. 1h), with real-time data from the buoy showing *H*_*m0*_ > 0.5 m, *T*_*m01*_ of 2-4 s and WNW wave directions (i.e., waves approaching from within the estuary rather than from offshore). Concurrent swell conditions measured at the Sydney offshore wave buoy (Fig. 1) were benign (<1 m with low-moderate period) and a nearby anemometer at Kurnell (10 km north-east of Deeban) recorded average wind speeds exceeding 30 knots with gusts over 40 knots. Surface wind speeds at the Deeban buoy (inferred by Sofar Ocean from the calculated wave spectra) were 15–20 knots. Throughout that period, wave data recorded by the Deeban Spotter buoy was unaffected, with no spikes in parameter values associated with erroneous displacement. Our observations follow others^48–51,61,63^ in suggesting that Datawell and Spotter GNSS wave buoys can suffer displacement measurement errors due to saltwater obstruction of satellite receivers, which may compromise derived spectra and parameters. Our analysis suggests that conditions with short-sea waves over an underlying swell may increase the potential for wave overtopping of buoys due to irregular buoy motion, short-wave collision and increased wave breaking.

The Spotter buoy appears to be more robust to displacement artefacts caused by satellite data disruption than the Datawell buoy. This is evident in only 7 fail Spotter data points during the buoy comparison at Old Bar compared to 24 fail Datawell data points (Fig. 5). Spotter buoys are not immune from displacement artefacts, however. In the full 5 months of Spotter data collected at Old Bar B there were 25 fail data points, with most causing spikes in peak parameters and some also causing spikes in mean parameters (Fig. 3). Whereas artefacts in Datawell displacement data typically cause unrealistic spikes in peak parameters (*T*_*p*_, θ_*p*_), mean parameters (*H*_*m0*_, *T*_*m02*_, θ_*m*_) are less conspicuously affected, but evidently deviate from the unaffected Spotter data. This is most evident during the storm wave conditions measured during 5–16 October in the buoy comparison deployments (Fig. 5). While artefacts in Spotter data are less common, they may cause prominent spikes in both peak and mean parameters, as occurred at Old Bar B on 4 January and 4 February 2019 (Fig. 3). Inspection of displacement data from the affected timestamps indicates that large spikes in mean parameters occurred when the amplitudes of artefacts were much higher (e.g., 20 times) than the average heave displacements recorded during the affected half hour. In comparison, artefacts in Datawell displacement data were usually only 2–5 times higher than average heave displacements.

We filter the displacement data recorded by both buoys using both a coarse spike filter and Butterworth filter to remove or minimise the influence of artefacts on derived wave spectra and parameters (see Methods, Data processing). Datawell also provides a manually operated Gap Repair tool to manage the issue, which uses an alternative digital filtering method^48^. We compared filtered displacement data and derived wave spectra following our methods with the same following application of the Datawell Gap Repair tool, comparing with concurrent Spotter data without artefacts as a benchmark. Our automated filtering methods provided equivalent or better results in removing or minimising displacement artefacts compared to application of the manually operated Gap Repair tool. The remnant influence of displacement artefacts are most evident in our processed parameter time series data as prominent spikes in peak parameters that occur more frequently in Datawell buoy data, although mean parameter values may also be affected in such instances. Our diagnostic tests described in the Methods (Quality control) identify all data points that may remain affected by displacement artefacts after data processing. Data users are advised to review suspect and fail data (using *Qflag* and *Qcode* values described in Table 5) and use such data with caution.

### Data loss and omission

Minor data gaps in the parameter time series data tables (*Qflag/Qcode* 9) occur during deployment service visits when the wave buoy and mooring are removed from the water for a short period of time (1–2 hours) to retrieve onboard data and either clean or swap the buoy and mooring. Such occasions are documented in the deployment readme files and are evident in the data tables as a change in buoy serial number if the instrument was replaced. Minor data loss may also occur during periods of limited satellite availability or if the satellite receiver experiences issues. The Maroubra deployment, for example, includes a 46-hour period during which the buoy did not record any data at all.

More significant data loss has occurred in some instances due to buoy or mooring failure, or when service visits were delayed for operational reasons. For Datawell deployments, the battery life limitation (c. 30 days) resulted in some moderate data gaps of several days due to delayed service visits. A data gap of 15 days occurred in the Bronte deployment when a Datawell buoy malfunctioned mid-deployment and failed to record any more data. The same instrument ceased to function during the Fairy Meadow deployment resulting in 33 days of data loss there relative to the concurrent Woonona deployment (Table 1).

If the deployment position changed significantly between service deployments (particularly water depth), or if there was an extended break (months) during the deployment, a location may have multiple data packages corresponding to successive interrupted deployments at the same location. Some notable examples are described below. A Spotter buoy at Boomerang A (Fig. 1d) was initially deployed in 11.4 m water depth and the buoy broke free from its mooring during a storm on 4/6/2019 (DD/MM/YYYY) and washed up on the beach. A replacement buoy was deployed 50 days later in deeper water (13.3 m) and that deployment was named Boomerang A2 (Table 2). Similarly, the service visit of the concurrent Datawell buoy deployment (Table 1) at Boomerang B (Fig. 1d) was delayed after the loss of the Spotter at Boomerang A, resulting in a data gap of 36 days after the Datawell batteries ran flat during which the mooring fouled (Fig. 2c). A Spotter buoy was redeployed on 24/7/2019 and that deployment was named Boomerang B2 (Table 2). That mooring eventually dragged its anchor in strong currents on 13/1/2020 and travelled 2 km south, and the four days of compromised data have been omitted from that data record.

The Collaroy location also consists of multiple data packages. The initial Collaroy deployment was from 2/3/2016 to 17/5/2016 (Collaroy 1), concurrent with the Narrabeen deployment. A Datawell buoy was redeployed at Collaroy on 3/6/2016 in a similar location (Collaroy2), prior to a severe storm^12^, and was retrieved on 21/7/2016 (Table 1). A Spotter buoy was later deployed at a similar location on 21/10/2019, which was dragged out of position during a storm on 9/2/2020 and retrieved 7 weeks later on 31/3/2020 in a heavily fouled condition (Collaroy3). Seven weeks of data collected after the storm have been omitted due to significant change in the position (and water depth) of the mooring (Table 2), and heavy fouling that was deemed to influence buoy behaviour after the storm. A replacement Spotter buoy was deployed on 31/3/2020 (Collaroy 4) and was maintained for more than 1.5 years. Additional deployments (Collaroy 5 and Collaroy 6) have been made since with brief gaps between (Tables 2, 3).

For Spotter deployments, a delayed service visit is only problematic if the motion of the buoy becomes compromised due to biofouling, or the solar panels are compromised by growth and the battery runs flat. Both impairments were the case during the first service deployment at Merimbula (Table 2), where the last month of data (3/4/2021 to 10/5/2021) has been omitted from the dataset following our assessment that buoy motion was compromised during that period by heavy fouling from blue mussels (Fig. 2d). In that case, the service visit had been delayed (>6 months from initial deployment) and biofouling was unprecedented in our experiences due to a local abundance of blue mussels in the region. Indeed, the Broulee and Bengello buoys (Fig. 1i) had been deployed a week prior to the Merimbula buoy, and when visited a week after retrieval of the Merimbula buoy, both moorings and buoys at those sites were functioning normally with considerably less biofouling. Finally, a location may have multiple numbered data packages to enable the delivery of retrieved data from an ongoing deployment (e.g., Bengello, Collaroy).

## Usage Notes

Many applications of the wave buoy data may benefit from complementary datasets including high-resolution seabed bathymetry and landform (geomorphology) mapping, and deep-water wave data from the offshore wave buoy network (Fig. 1). Seabed bathymetry and landform mapping data cover the locations of all nearshore wave buoy deployments and will be useful for interpreting the nearshore wave data and for application in shallow-water wave models (e.g., bathymetry and bottom friction). These data are described below.

The *Australian Wave Buoy Operations and Data Management Guidelines*^44^ are available from Ocean Best Practices and provide further details on buoy deployment and wave data quality control methods used in our program. Additional information on wave buoy deployments and wave modelling tools from the *NSW Nearshore Wave Data Program* are available online^24^.

### Coastal seabed bathymetry and landforms mapping data

A seamless topography-bathymetry model covering the entire NSW coastline may also be of interest to users of the wave buoy data. The model provides 5-m spatial resolution elevation data extending from >200 m distance inland out to 30 m water depth on average (49 m maximum). The dataset derives from seamless airborne lidar topography-bathymetry surveys carried out during July-December 2018^17^. Data covering the entire NSW coast can be browsed in an online Geocortex viewer and downloaded through the NSW SEED portal^14^.

The lidar bathymetry data have also been classified using spatial analysis techniques to derive detailed mapping of seabed landform features, including rocky reefs and sediment plains^16,74^. The seabed landforms mapping data can be browsed in an online Geocortex viewer and downloaded from the NSW SEED portal^15^.

Additional to lidar-derived seabed mapping covering the whole NSW coast, vessel-based multibeam echosounder surveys are being carried out to capture high-resolution coastal bathymetry data to 50–60 m water depths in selected sediment compartments^17^. Bathymetry and seabed landforms mapping data from those surveys is also available from the NSW SEED portal^75^.

### Offshore wave buoy data

Long-term offshore wave buoy deployments have been maintained at seven locations along the NSW coast for multiple decades^13^ (Fig. 1). Deep-water wave data from the offshore wave buoy network are available from the Australian National Wave Archive^59^ or by contacting Manly Hydraulics Laboratory.

## Data Availability

All data preparation, processing and analysis, and the generation of plots and data tables were carried out using MATLAB. The code used to process buoy data from displacements to the wave parameter time series data presented here is provided with the data packages on the SEED portal in html format and can be viewed as they appear in MATLAB by anyone without access to that proprietary software.
